# Copy Number Variation Analysis of Euploid Pregnancy Loss

**DOI:** 10.3389/fgene.2022.766492

**Published:** 2022-03-23

**Authors:** Chongjuan Gu, Huan Gao, Kuanrong Li, Xinyu Dai, Zhao Yang, Ru Li, Canliang Wen, Yaojuan He

**Affiliations:** ^1^ Department of Obstetrics and Gynecology, Guangzhou Women and Children’s Medical Center, Guangzhou Medical University, Guangzhou, China; ^2^ Department of Toxicology, School of Public Health, Sun Yat-sen University, Guangzhou, China; ^3^ Institute of Pediatrics, Guangzhou Women and Children’s Medical Center, Guangzhou Medical University, Guangzhou, China; ^4^ School of Life Sciences, South China Normal University, Guangzhou, China; ^5^ West China Hospital, Sichuan University, Chengdu, China; ^6^ Prenatal Diagnostic Center, Guangzhou Women and Children’s Medical Center, Guangzhou Medical University, Guangzhou, China

**Keywords:** copy number variant, products of conception, pregnancy loss, chromosomal array, bioinformatics, genome, fetal death

## Abstract

**Objectives:** Copy number variant (CNV) is believed to be the potential genetic cause of pregnancy loss. However, CNVs less than 3 Mb in euploid products of conceptions (POCs) remain largely unexplored. The aim of this study was to investigate the features of CNVs less than 3 Mb in POCs and their potential clinical significance in pregnancy loss/fetal death.

**Methods:** CNV data were extracted from a cohort in our institution and 19 peer-reviewed publications, and only those CNVs less than 3 Mb detected in euploid pregnancy loss/fetal death were included. We conducted a CNV map to analyze the distribution of CNVs in chromosomes using R packages karyoploteR_1.10.5. Gene names and annotated gene types covered by those CNVs were mined from the human Release 19 reference genome file and GENECODE database. We assessed the expression patterns and the consequences of murine knock-out of those genes using TiGER and Mouse Genome Informatics (MGI) databases. Functional enrichment and pathway analysis for genes in CNVs were performed using clusterProfiler V3.12.0.

**Result:** Breakpoints of 564 CNVs less than 3 Mb were obtained from 442 euploid POCs, with 349 gains and 185 losses. The CNV map showed that CNVs were distributed in all chromosomes, with the highest frequency detected in chromosome 22 and the lowest frequency in chromosome Y, and CNVs showed a higher density in the pericentromeric and sub-telomeric regions. A total of 5,414 genes mined from the CNV regions (CNVRs), Gene Ontology (GO), and pathway analysis showed that the genes were significantly enriched in multiple terms, especially in sensory perception, membrane region, and tight junction. A total of 995 protein-coding genes have been reported to present mammalian phenotypes in MGI, and 276 of them lead to embryonic lethality or abnormal embryo/placenta in knock-out mouse models. CNV located at 19p13.3 was the most common CNV of all POCs.

**Conclusion:** CNVs less than 3 Mb in euploid POCs distribute unevenly in all chromosomes, and a higher density was seen in the pericentromeric and sub-telomeric regions. The genes in those CNVRs are significantly enriched in biological processes and pathways that are important to embryonic/fetal development. CNV in 19p13.3 and the variations of *ARID3A* and *FSTL3* might contribute to pregnancy loss.

## Background

Approximately 15–20% of clinically recognized pregnancies end in pregnancy loss (Practice Committee of the American Society for Reproductive Medicine, 2012; ESHRE Guideline Group on RPL et al., 2018), and the etiology is complicated. It is evident that there are many genetic and environmental factors that are essential for a successful pregnancy, and disruption of any of them could cause pregnancy loss (Yamada et al., 2005). From the genetic perspective, abnormal number and structure of chromosomes are clearly pathogenic genetic causes, and smaller copy number variant (CNV) and mutations in genes that are important for early fetal development are also the potential genetic causes (Colley et al., 2019).

The array-based detection has been used to detect the chromosomal abnormalities of pregnancy loss owing to its higher resolution and detection rates (Hillman et al., 2011; Dhillon et al., 2014). Meanwhile, the array-based detection allows unbiased search for CNVs across the whole genome, which involves unbalanced rearrangements that increase or decrease the DNA content. CNV is associated with a wide range of human diseases, including congenital anomalies and neurodevelopmental disorders (Grayton et al., 2012; Wapner et al., 2012; Dong et al., 2016). However, owing to limited data, it is challenging for clinicians and geneticists to interpret CNVs detected in POCs. Those “pathogenic CNVs” are based on individuals with neurodevelopmental disorders and/or congenital anomalies or fetuses with ultrasound abnormalities (Riggs et al., 2020), as well as on healthy population [e.g., Database of Genomic Variants (DGV) and the 1,000 Genomes database] (Lee and Scherer, 2010; MacDonald et al., 2014), which cannot accurately interpret CNVs in demised embryos/fetuses. There is a continuous spectrum of phenotypic effects of CNV, varying from adaptive and maladaptive traits to embryonic lethality (Beckmann et al., 2007; Hurles et al., 2008). Most of the CNVs less than 3 Mb have been believed not to be associated with adverse phenotypes among healthy individuals (Zarrei et al., 2015). However, the roles of these CNVs less than 3 Mb played in pregnancy loss remain largely unexplored. We suppose that some of the small-sized CNVs detected in POCs involving embryonic lethal or placental function-specific genes have never been reported in DGV and might contribute to pregnancy loss/fetal death.

To further understand the features of CNVs less than 3 Mb detected in POCs and potential clinical roles of those CNVs in euploid pregnancy loss, we constructed a CNV map based on the data obtained from our samples and reported in the literature and analyzed the gene content and function *in silico*.

## Materials and Methods

### Cohort Copy Number Variant Data

The first part of CNV data was extracted from a retrospective, hospital-based cohort of the Guangzhou Women and Children’s Medical Center, a tertiary referral hospital in South China. The study protocol was approved by the Ethics Committee of the institute (2020-15001). All patients provided a written informed consent for the tests and the inclusion of results in research. All women were Han Chinese who experienced clinically confirmed pregnancy loss or fetal death according to the guideline (Doubilet et al., 2013) and underwent chromosomal microarray analysis (CMA) detection of the fresh POC sample in our hospital. The methods used for DNA extraction, maternal cell contamination test, and CMA platform have been reported in our previous publication (Gu et al., 2021). The reporting threshold of the copy number result was set at 100 kb with marker count ≥50 bp. Data were visualized and analyzed with the Chromosome Analysis Suite (ChAS) software (Affymetrix, Santa Clara, CA) based on the GRCh37/hg19 assembly. In this study, only those euploid POCs with CNV size less than 3 Mb were included.

### Published Copy Number Variant Data and Quality Control

The second part of CNV data was extracted from the peer-reviewed publications. The literature search was focused on studies using microarrays and next-generation sequencing (NGS) to detect POC following pregnancy loss or fetal death. PubMed, Medline, Embase, and CNKI databases were searched electronically, with the last search updated on 30 September 2020. The complete search string is outlined in Supplementary Table S1. Data included in this study must meet the following criteria: 1) the subjects of the study were POCs of pregnancy loss or fetal death; 2) the methods of detection were genome-wide assessment and estimated breakpoint resolution. Studies or data would be excluded if the chromosomal karyotype was aneuploid or if the CNV length was longer than 3 Mb. Study selection was achieved independently by two investigators by screening the title, abstract, and full-text. The data of the eligible studies were documented in a table detailing the methods of the detection, chromosomal locations of CNV, sites of CNV beginning and end, and CNV gain or loss, etc. Then, quality control was performed independently by two investigators.

All CNV data were reported in the hg19 version except for two studies. In one study (Donaghue et al., 2017), CNVs were shown in OMIM, and we obtained the location information and converted it to the hg38 version according to the OMIM ID (https://omim.org/). Together with another study (Rajcan-Separovic et al., 2010), in which CNVs were also reported in the hg38 version, we converted CNV coordinates into the human assembly hg19 using the UCSC liftOver tool 18 (http://genome.ucsc.edu/cgi-bin/hgLiftOver).

### Generating the Copy Number Variant Map

To capture the maximum extent of CNVs, we combined the data from our cohort and the published data into a single map. First, we analyzed the density distribution of CNVs through locating all CNVs to the chromosomes using R packages karyoploteR_1.10.5. Second, we investigated the distribution of the CNVs in the pericentromeric and sub-telomeric regions of the genome. We used a sliding window of 5 Mb with steps of 0.5 Mb within 18 Mb from both sides of the centromeres (9 Mb from each side) and 9 Mb away from the telomeres. The percentage of un-gapped nucleotides varying in each window was calculated per chromosome and plotted for all chromosomes. The chromosome length information and telomere and centromere position file was obtained from the UCSC database (hg19). The R packages ggplot2_3.3.0 were used to construct the histograms. Third, to explore the differences between CNVs detected in POCs and CNVs reported in human diseases, we compared the CNVs including those with data in the Database of Genomic Variants (DGV, http://dgv.tcag.ca/dgv/docs/GRCh37_hg19_variants_2020-02-25.txt), the CNVs reciprocal overlap more than 75% with the CNVs in DGV, and have the correspondent gain or loss which were considered reported CNV.

### Copy Number Variant Gene Content and Gene Characteristics

Gene names and chromosomal coordinates of CNVs were mined from the human Release 19 reference genome file (https://www.gencodegenes.org/human/release_19.html), and CNV location information in the GENECODE database using the bedtools version 1.58 intersects function to investigate CNVRs coverage genes and annotate gene types. In order to explore the functional relevance of CNVs, we assessed the expression patterns of their integral genes using the TiGER database (Tissue-specific Gene Expression and Regulation: http://bioinfo.wilmer.jhu.edu/tiger/) (Liu et al., 2008) and the consequences of murine knock-out studies using the Mouse Genome Informatics (MGI) database (http://www.informatics.jax.org). Then, we focused on the genes expressed in the placenta and the genes resulted in embryonic lethality and abnormal embryonic development in knock-out murine.

### Functional Gene Enrichment Analyses

Functional enrichment and pathway analysis for protein-coding genes in CNVs was performed using the clusterProfiler V3.12.0 R package 19. Gene-enrichment for Kyoto Encyclopedia of Genes and Genomes (KEGG) pathways and GO terms (biological process, cellular component, and molecular function) were carried out for gain or loss CNV groups separately and together. A *p* value < 0.05 was considered statistically significant for GO terms and pathway analysis. Data were reported as significantly enriched GO terms and pathways.

## Results

### Characteristics of Copy Number Variant Data

A total of 564 CNVs less than 3 Mb (mean: 690.2 Kb, ranging from 6.4 Kb to 2.98 Mb) were obtained from 442 euploid POCs, of which 176 CNVs were detected in our institution and 266 were extracted from 19 peer-reviewed publications (Supplementary Figure S1). All CNVs were detected using SNP array (9 research studies) (Reddy et al., 2012; Kooper et al., 2014; Wang et al., 2017; Qi et al., 2018; Zhu et al., 2018; Mao et al., 2019; Sato et al., 2019; Yang et al., 2019; Wang et al., 2020), array CGH (4 research studies) (Shimokawa et al., 2006; Deshpande et al., 2010; Rajcan-Separovic et al., 2010; Donaghue et al., 2017), or CMA (6 research studies plus our data) (Sahlin et al., 2014; Wang et al., 2014; Rosenfeld et al., 2015; Parchem et al., 2018; Chau et al., 2020; Zhang et al., 2021). The threshold of those studies called the CNVs has been reported ranging from 50 to 135 Kb. Among the 19 peer-reviewed publications, 11 were based on the report of 141 Caucasian cases in total and 8 based on the report of 125 Asian cases in total. Among the 564 CNVs, gains (microduplications) were largely more than losses (microdeletions, 349 vs. 185), and 30 CNVs were uncertain gain or loss from the articles. After removing the repeated CNVs and 30 CNVs unknown gain or loss, we compared the CNVs in this study with DGV data, and the results showed that 234 (52%) variants were reported, while 215 (48%) variants were not reported by DGV (Table 1).

**TABLE 1:** CNV data from our institution and 19 peer-reviewed publications.

	Total	Gain	Loss	Unknown
Total	564	349	185	30
Our hospital	264	188	76	0
Published publications	300	161	109	30
Reported in DGV^1^	235	161	74	
Unreported in DGV^1^	244	129	85	

1 After removing the same CNVs in different cases.

DGV, Database of Genomic Variants.

### Distribution of Copy Number Variant in Chromosomes

**FIGURE 1:**
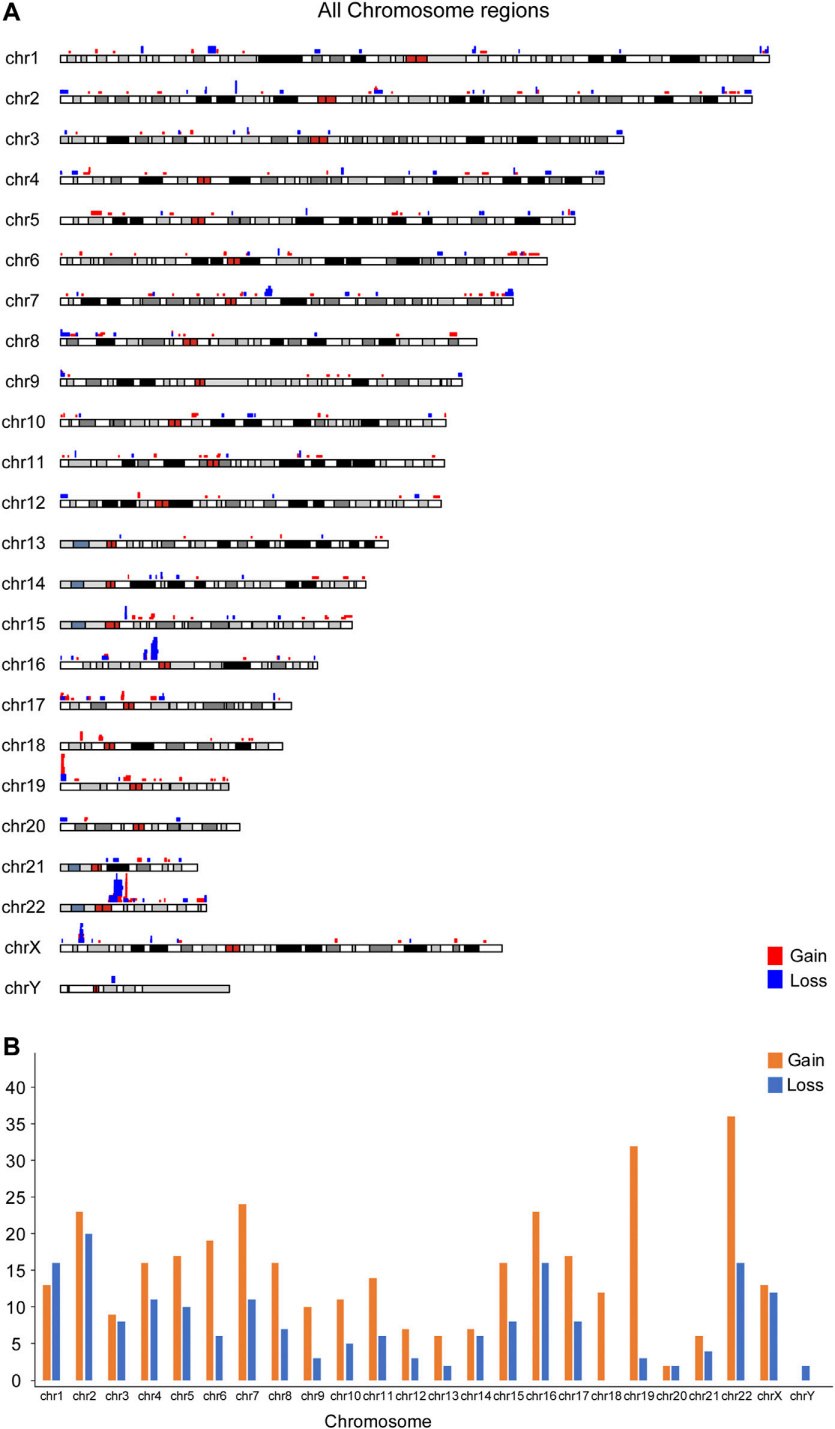
Chromosomal distribution of 564 CNVs less than 3 Mb from 422 euploid pregnancy loss/fetal death. **(A)** Overall map for the CNVs; **(B)** CNV number in each chromosome.

**FIGURE 2:**
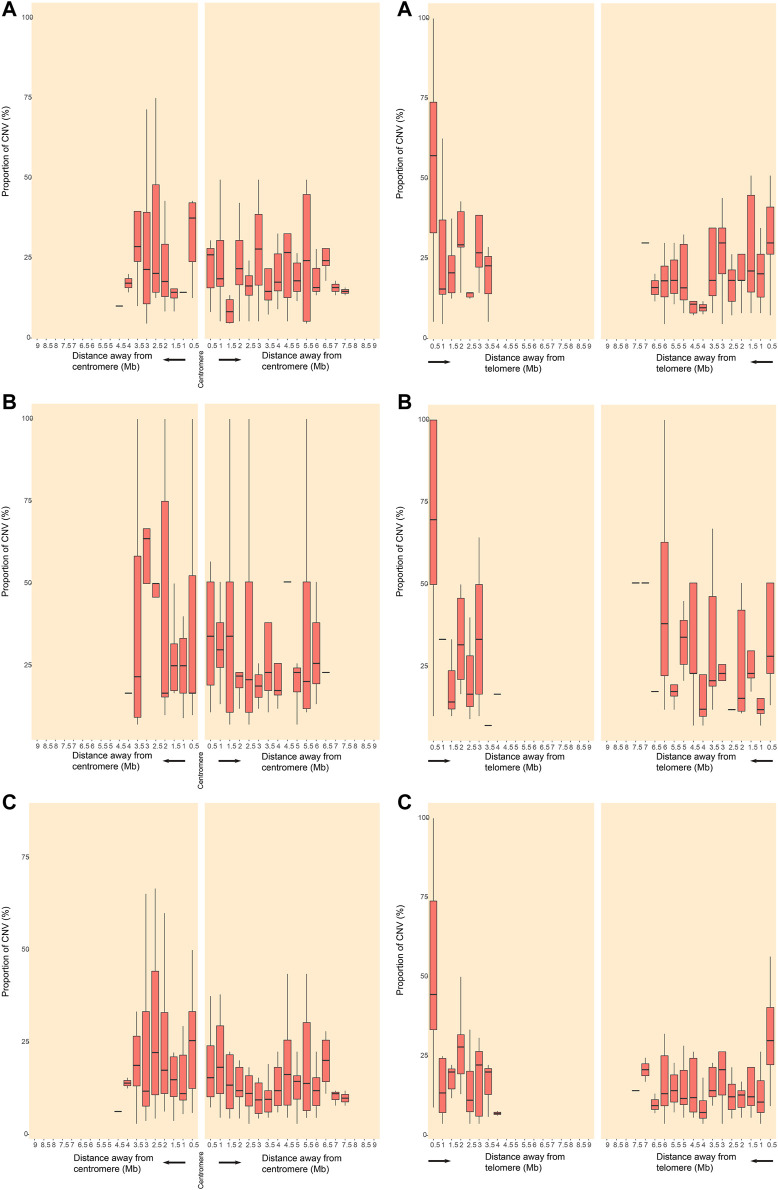
Distribution of CNVRs in pericentromeric and sub-telomeric regions of human chromosomes. CNVRs gains **(A)**, CNVRs losses **(B)**, and CNVRs gains and losses in the inclusive map **(C)** are shown for pericentromeric regions (left panels) and sub-telomeric regions (right panels). The y axes indicate the percentage of nucleotides in each window that may involve CNVs.

The location and the number of all CNVs (564) for euploid POCs on chromosomes are shown in Figure 1. We investigated the CNVs in genomic gains and losses independently and also merged the two versions to generate a consensus map that represents all variations (Figure 1A). CNVs were found in all chromosomes, and the number varied from 2 CNVs in chromosome Y to 53 CNVs in chromosome 22. For gain, chromosome 22 showed the highest number (36 CNVs), followed by chromosome 19 (32 CNVs). Chromosome Y showed no CNVs gain, and chromosome 20 showed only 2 CNVs gain (Figure 1B). For losses, chromosome 2 showed the highest number of 20 CNVs, followed by chromosome 1, chromosome 16, and chromosome 22 that showed 16 CNVs, respectively. There was no CNV loss on chromosome 18, and only 2 CNVs loss on chromosome 20 (Figure 1B). Figure 2 illustrates the distribution of CNVs in the pericentromeric and sub-telomeric regions, showing that the pericentromeric regions have a higher proportion of CNVs and the same characteristics are observed in both gain and loss in the sub-telomeric regions.

### Functional Enrichment

**FIGURE 3:**
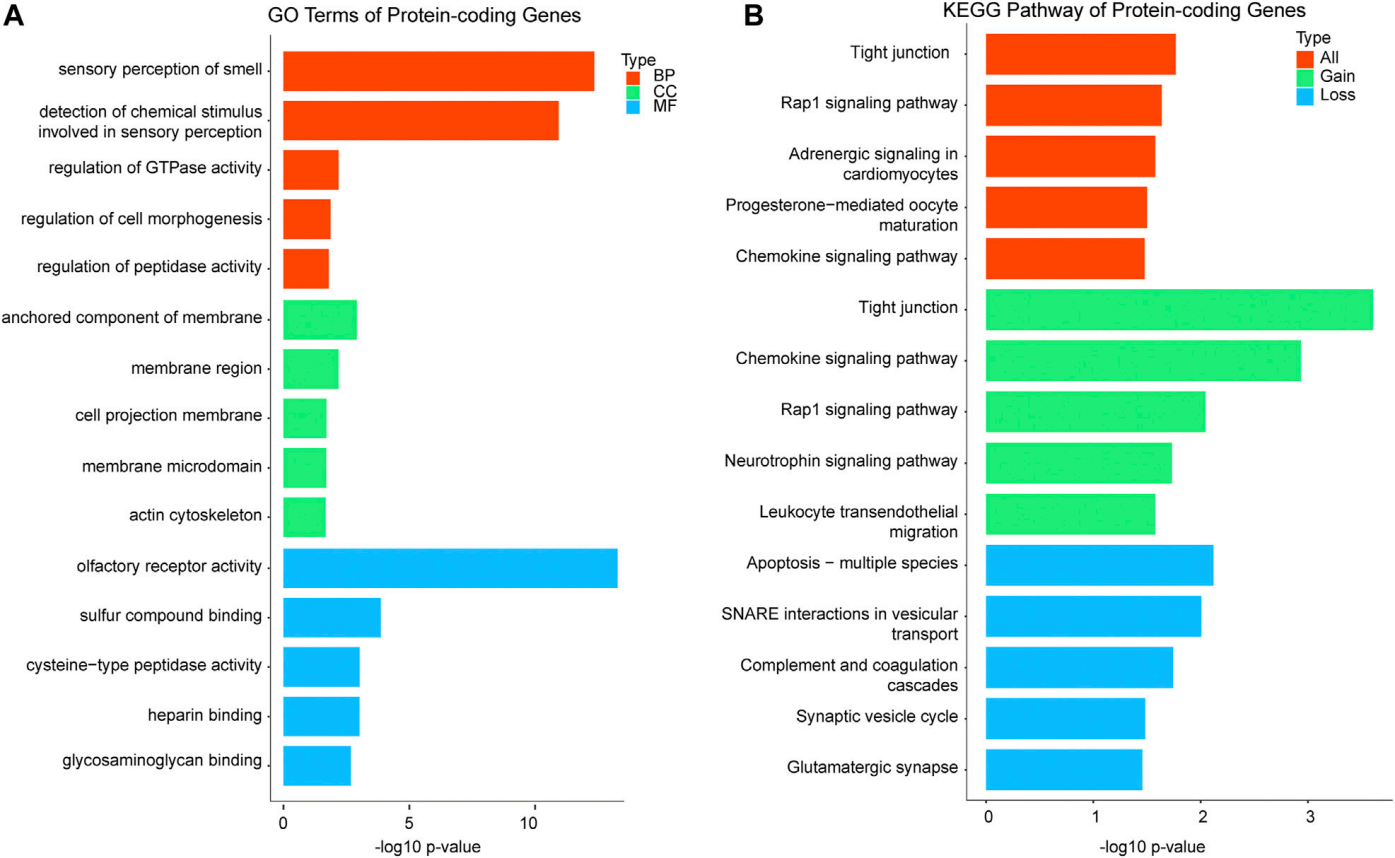
Functional enrichment analysis of significant terms. A total of 1,862 protein-coding genes were submitted to clusterProfiler. **(A)** Top GO terms enriched for BP, CC, and MF in protein-coding genes; GO terms are assigned to y-axis, and negative log10 *p* values are assigned to x-axis; **(B)** top KEGG pathways for protein-coding genes of gains, losses, and both, and KEGG terms are assigned to y-axis, and negative log10 *p* values are assigned to x-axis; GO, gene ontology; KEGG, Kyoto Encyclopedia of Genes and Genomes; BP, biological process; CC, cellular component; MF, molecular function.

After removing the same CNVs in different cases, 479 CNVRs remained, including 291 gains, 159 losses, and 29 CNVRs uncertain gains or losses. A total of 5,414 genes including 1,862 protein-coding genes and 1,284 noncoding genes (the categories of the 5,414 genes are shown in Supplementary Figure S2) were mined from the 479 CNVRs. GO and KEGG analyses of the involved protein-coding genes were performed. For each GO analysis domain, the five top most significantly (*p* value < 0.05) enriched GO terms are presented in Figure 3. The genes in GO biological process were primarily associated with “sensory perception of smell,” “detection of the chemical stimulus involved in sensory perception,” and “regulation of gtpase activity.” The genes in the GO cellular component were mostly enriched in “anchored component of the membrane,” “membrane region,” and “cell projection membrane.” The genes in GO molecular function were mainly associated with “olfactory receptor activity,” “sulfur compound binding,” and “cysteine-type peptidase activity” (Figure 3A). The protein-coding genes KEGG pathway analysis indicated that the CNVRs were intensively associated with “tight junction,” “Rap1 signaling pathway,” “adrenergic signaling in cardiomyocytes,” “progesterone-mediated oocyte maturation,” and “chemokine signaling pathway” (Figure 3B). The details of GO terms and KEGG pathways are shown in Supplementary Table S2.

### Gene Characteristics

**FIGURE 4:**
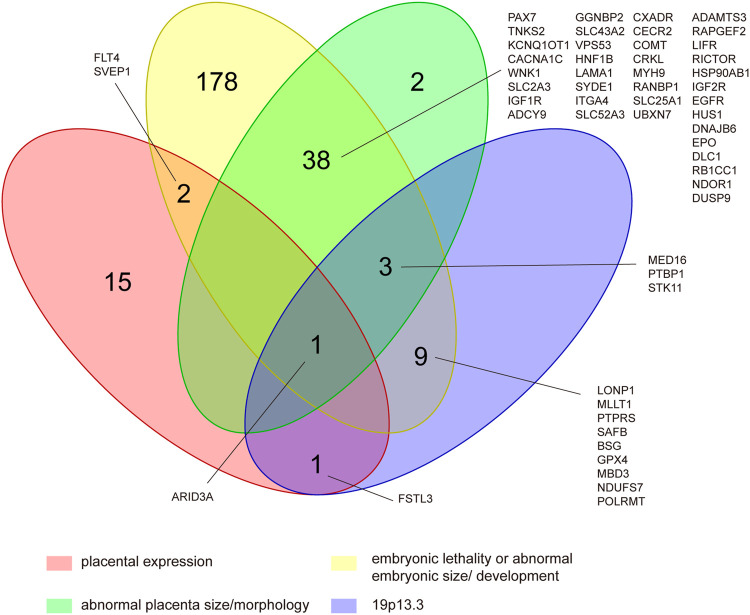
Identification of CNV genes in POCs associated with embryonic lethality or abnormal embryonic size/development, abnormal placental size/morphology, and placental expression located in 19p13.3. This was determined by assessing 995 protein-coding genes of the CNVRs that had reported to present mammalian phenotypes in mouse knock-out studies and cataloged on MGI as well as assessing 19 human placental-expressed genes listed on TiGER.

Among the 1,862 protein-coding genes, 53% (995/1862) of them have been reported to present mammalian phenotypes in MGI. The number of genes that results in embryonic lethality or abnormal embryonic size/development and abnormal placental size/morphology in knock-out models were 233 and 44, respectively (Figure 4). The results of tissue-specific expression analysis of protein-coding genes showed that 19 genes were placental-specific or placental expression. The details of the involved genes and CNVs are shown in Supplementary Table S3. The most frequent CNV was located on 19p13.3, which was detected in 11 POCs with 9 gains and 2 losses, with a size ranging from 523.9 Kb to 1.5 Mb (Supplementary Table S3). Among CNVRs in 19p13.3, 13 genes with mammalian phenotypes in MGI caused murine embryonic lethality or abnormal embryonic/placental size/morphology in knock-out models, and 2 genes showed placental expression (Figure 4 and Supplementary Table S3).

## Discussion

This study presents a unique analysis of CNVs less than 3 Mb detected in euploid POCs and their integral gene content in a large cohort and 19 published studies in order to evaluate their overall chromosomal distribution, genomic features, and functions based on bioinformatics. Collectively, all the chromosomes are susceptible to CNV in POCs, and CNVs distribute unevenly along the chromosomes and among chromosomal regions. Some CNVs might have a pathogenic role in pregnancy loss because of containing embryonic lethality genes.

Gene and segmental duplications are thought to have a significant role in gene and genome evolution and are often under positive selection, whereas deletions are biased away from certain categories and more likely to cause disease or alter the fitness (Hurles, 2004; Redon et al., 2006; Uddin et al., 2014). In our data, there are more gains than losses detected in euploid POCs (349 vs. 185), which is in contrary to CNVs in healthy individuals from various populations according to the research of Mehdi Zarrei, who analyzed 23 studies and reported that the losses were almost 10 times the gains (Redon et al., 2006). The mechanism of germ line CNVs is complicated, and it is unclear that the proportion of duplication and deletion is in the early stage of embryogenesis. There are approximately 22% of spontaneous conceptions ending in biochemical pregnancy losses (BPLs), which are poorly understood since embryonic arrest is prior to the development of a clinical pregnancy (Wilcox et al., 1988; Ellish et al., 1996; Zinaman et al., 1996). If the variational chances to duplication and deletion in embryogenesis are equal, it is possible that the embryo with CNV duplication might be more likely to cause pregnant failure than the embryo with CNV deletion.

In our data, CNVs distribute unevenly in all chromosomes, and chromosome 22 is found to have the highest variability, which is consistent with CNVs in healthy individuals (Makino et al., 2013; Zarrei et al., 2015). However, Y chromosome carries the lowest number of CNVs in our study, which is contrary to the highest proportion of CNVs in Y chromosome reported in healthy individuals (Zarrei et al., 2015). Those results indicate that the pregnancy with Y chromosome microduplications or microdeletions might not result in embryonic/fetal death. After all, the biological function of Y chromosome is believed to mainly impact male fitness such as fertility (Quintana-Murci et al., 2001; Schlegel, 2002). Our study also demonstrates that CNVs unevenly distribute within the chromosome. The pericentromeric and sub-telomeric regions have a higher density of CNVs, in both gain and loss, which are same as the results of healthy individuals (Zarrei et al., 2015).

For gene functional enrichment, to our surprise, the two most significant GO germs in biological process of the protein-coding genes are involved in “sensory perception of smell” and “detection of the chemical stimulus involved in sensory perception”. Sensory development is complex, with both morphological and neural components (Clark-Gambelunghe and Clark, 2015). The tissues of the oral cavity, eye, and auditory system form the face and palate between 6 and 12 gestational weeks (Witt, 2019). The development of the nervous system and sensory perception is established throughout the fetal and postnatal period, which is important for fetal survival. We speculate that genes involved in sensory perception might be dose-sensitive and have potential to cause embryonic arrest when CNV occurs. In GO cellular component, four of top five significant terms are enriched in membrane-related components, such as “anchored component of the membrane,” “membrane region,” and “membrane microdomain.” The genes for the cell membrane component are vital to embryonic development, and our results imply that functions of those genes might be easily affected by gene dosage. The genes in GO molecular function are significantly associated with olfactory receptor activity, sulfur compound binding, and heparin binding, and those functions are related to transmembrane transport. Our results also show that the KEGG pathway is significantly related to “tight junction.” It is well known that the tight junction (TJ) is an essential component of the differentiated epithelial cell required for polarization and intercellular integrity during early development (Eckert and Fleming, 2008; Green et al., 2019). These results indicate that pregnancy with CNVs involving genes in membrane component, transmembrane transport, and TJ might relate to developmental arrest.

Among the protein-coding genes, 276 genes showed embryonic lethality or abnormal embryonic/placental size/morphology in knock-out mouse models. Theoretically, pregnancy with CNV carrying those genes could increase the risk for embryonic demise, which, however, needs to be confirmed by further studies. In addition, CNV located at 19p13.3 is found to be the most frequent one, in 11 POCs. It is interesting in our results that all the 13 genes contained in 19p13.3 that have mammalian phenotypes in MGI are shown to cause murine embryonic lethality or abnormal embryonic/placental size/morphology in the knock-out model. Chromosome 19 has the highest gene density of all human chromosomes (Grimwood et al., 2004), and CNVs in 19p13.3 have been reported in several patients with intellectual disability and congenital malformations (Orellana et al., 2015; Palumbo et al., 2016). Our study suggests that CNV in 19p13.3 might be pathogenic in pregnancy loss/fetal death.

Among those genes included in 19p13.3, two placental-expressed genes are worth of attention, namely AT-rich interaction domain 3A (*ARID3A*) and follistatin-like 3 (*FSTL3*). *ARID3A* has been reported essential to the execution of the first cell fate decision and is of importance to regulate mesoderm differentiation and nephric tubule regeneration in animal models and has a vital role in placental development (Rhee et al., 2015; Popowski et al., 2017; Suzuki et al., 2019). *FSTL3* has been demonstrated to be expressed on the maternal–fetoplacental interface in the first trimester and regulates the invasion and migration of trophoblast, which is important for establishing and maintaining normal pregnancy (Xie et al., 2018; Founds and Stolz, 2020; Xu et al., 2020). In addition, arid3a and fstl3 show abnormal phenotypes of multiple organs in knockout mouse models. Therefore, it is possible that duplication or deletion in *ARID3A* and *FSTL3* results in embryonic/fetal development arresting.

Our study has several limitations. First, CNV data were extracted from our laboratory and published studies, which were detected by different platforms, so that the potential methodological bias cannot be eliminated. Second, our study did not compare CNVs in POCs from pregnancy loss/fetal death with healthy controls, and therefore cautions should be taken in the interpretation of the pathogenic CNVs in pregnancy loss/fetal death. Third, we were unable to identify those CNVs in parents or to achieve those data about parental origin, which affects the determination of pathogenic CNV to a certain extent, especially for pathogenicity of 19p13.3.

In conclusion, this study shows that CNVs less than 3 Mb in euploid POCs distribute unevenly in all chromosomes and have a higher density in the pericentromeric and sub-telomeric regions. The CNVRs are significantly enriched in genes involving sensory perception, membrane-related components, and tight junction, and those biological processes and pathways are important for embryonic/fetal development. CNV in 19p13.3 might have a pathogenic role in pregnancy loss, and the variations of *ARID3A* and *FSTL3* might be a predisposing risk for pregnancy loss. A further study is needed to compare those CNVs with the control group and identify those CNVs in the parents for getting inheritance information.

## Data Availability

The raw data supporting the conclusion of this article will be made available by the authors, without undue reservation.

## Supplementary Material

Supplementary Figure S1 The flew chart of the search and selection of the peer-reviewed publications.

Supplementary Figure S2 The categories of the 5,414 genes.

Supplementary Table S1 Complete search used in the systematic literature search.

Supplementary Table S2 The details of GO terms and KEGG pathways.

Supplementary Table S3 The details of placental expression genes, genes with mammalian phenotypes in MGI caused murine embryonic lethality or abnormal embryonic/placental size/morphology, as well as genes in 19p13.3.

## References

[B1] Beckmann J. S. Estivill X. Antonarakis S. E. (2007). Copy Number Variants and Genetic Traits: Closer to the Resolution of Phenotypic to Genotypic Variability. Nat. Rev. Genet. 8, 639–646. 10.1038/nrg2149 17637735

[B2] ESHRE Guideline Group on RPL Bender Atik R. Christiansen O. B. Elson J. Kolte A. M. Lewis S. Middel, dorp S. (2018). ESHRE Guideline: Recurrent Pregnancy Loss. Hum. Reprod. Open 2018, hoy004. 10.1093/hropen/hoy004 31486805 PMC6276652

[B3] Chau M. H. K. Wang H. Lai Y. Zhang Y. Xu F. Tang Y. (2020). Low-pass Genome Sequencing: a Validated Method in Clinical Cytogenetics. Hum. Genet. 139, 1403–1415. 10.1007/s00439-020-02185-9 32451733

[B4] Clark-Gambelunghe M. B. Clark D. A. (2015). Sensory Development. Pediatr. Clin. North America 62, 367–384. 10.1016/j.pcl.2014.11.003 25836703

[B5] Colley E. Hamilton S. Smith P. Morgan N. V. Coomarasamy A. Allen S. (2019). Potential Genetic Causes of Miscarriage in Euploid Pregnancies: a Systematic Review. Hum. Reprod. Update 25, 452–472. 10.1093/humupd/dmz015 31150545

[B6] Deshpande M. Harper J. Holloway M. Palmer R. Wang R. (2010). Evaluation of Array Comparative Genomic Hybridization for Genetic Analysis of Chorionic Villus Sampling from Pregnancy Loss in Comparison to Karyotyping and Multiplex Ligation-dependent Probe Amplification. Genet. Test. Mol. Biomarkers 14, 421–424. 10.1089/gtmb.2010.0014 20408732

[B7] Dhillon R. Hillman S. Morris R. McMullan D. Williams D. Coomarasamy A. (2014). Additional Information from Chromosomal Microarray Analysis (CMA) over Conventional Karyotyping when Diagnosing Chromosomal Abnormalities in Miscarriage: a Systematic Review and Meta-Analysis. Bjog: Int. J. Obstet. Gy 121, 11–21. 10.1111/1471-0528.12382 23859082

[B8] Donaghue C. Davies N. Ahn J. W. Thomas H. Ogilvie C. M. Mann K. (2017). Efficient and Cost-Effective Genetic Analysis of Products of conception and Fetal Tissues Using a QF-PCR/array CGH Strategy; Five Years of Data. Mol. Cytogenet. 10, 12. 10.1186/s13039-017-0313-9 28396697 PMC5382376

[B9] Dong Z. Zhang J. Hu P. Chen H. Xu J. Tian Q. (2016). Low-pass Whole-Genome Sequencing in Clinical Cytogenetics: a Validated Approach. Genet. Med. 18, 940–948. 10.1038/gim.2015.199 26820068

[B10] Doubilet P. M. Benson C. B. Bourne T. Blaivas M. (2013). Society of Radiologists in Ultrasound Multispecialty Panel on Early First Trimester Diagnosis of Diagnostic Criteria for Nonviable Pregnancy Early in the First Trimester. N. Engl. J. Med. 369, 1443–1451. 10.1056/nejmra1302417 24106937

[B11] Eckert J. J. Fleming T. P. (2008). Tight junction Biogenesis during Early Development. Biochim. Biophys. Acta (Bba) - Biomembranes 1778, 717–728. 10.1016/j.bbamem.2007.09.031 18339299

[B12] Ellish N. J. Saboda K. O'Connor J. Nasca P. C. Stanek E. J. Boyle C. (1996). A Prospective Study of Early Pregnancy Loss. Hum. Reprod. 11, 406–412. 10.1093/humrep/11.2.406 8671233

[B13] Founds S. A. Stolz D. B. (2020). Gene Expression of Four Targets *In Situ* of the First Trimester Maternal-Fetoplacental Interface. Tissue and Cell 64, 101313. 10.1016/j.tice.2019.101313 32473702 PMC7264084

[B14] Grayton H. M. Fernandes C. Rujescu D. Collier D. A. (2012). Copy Number Variations in Neurodevelopmental Disorders. Prog. Neurobiol. 99, 81–91. 10.1016/j.pneurobio.2012.07.005 22813947

[B15] Green K. J. Jaiganesh A. Broussard J. A. (2019). Desmosomes: Essential Contributors to an Integrated Intercellular junction Network. F1000Res 8, F1000. 10.12688/f1000research.20942.1 PMC6944264 31942240

[B16] Grimwood J. Gordon L. A. Olsen A. Terry A. Schmutz J. Lamerdin J. (2004). The DNA Sequence and Biology of Human Chromosome 19. Nature 428, 529–535. 10.1038/nature02399 15057824

[B17] Gu C. Li K. Li R. Li L. Li X. Dai X. (2021). Chromosomal Aneuploidy Associated with Clinical Characteristics of Pregnancy Loss. Front. Genet. 12, 667697. 10.3389/fgene.2021.667697 33936179 PMC8083898

[B18] Hillman S. C. Pretlove S. Coomarasamy A. McMullan D. J. Davison E. V. Maher E. R. (2011). Additional Information from Array Comparative Genomic Hybridization Technology over Conventional Karyotyping in Prenatal Diagnosis: a Systematic Review and Meta-Analysis. Ultrasound Obstet. Gynecol. 37, 6–14. 10.1002/uog.7754 20658510

[B19] Hurles M. (2004). Gene Duplication: the Genomic Trade in Spare Parts. Plos Biol. 2, E206. 10.1371/journal.pbio.0020206 15252449 PMC449868

[B20] Hurles M. E. Dermitzakis E. T. Tyler-Smith C. (2008). The Functional Impact of Structural Variation in Humans. Trends Genet. 24, 238–245. 10.1016/j.tig.2008.03.001 18378036 PMC2869026

[B21] Kooper A. J. Faas B. H. Feenstra I. de Leeuw N. Smeets D. F. (2014). Best Diagnostic Approach for the Genetic Evaluation of Fetuses after Intrauterine Death in First, Second or Third Trimester: QF-PCR, Karyotyping And/or Genome Wide SNP Array Analysis. Mol. Cytogenet. 7, 6. 10.1186/1755-8166-7-6 24428858 PMC3906897

[B22] Lee C. Scherer S. W. (2010). The Clinical Context of Copy Number Variation in the Human Genome. Expert Rev. Mol. Med. 12, e8. 10.1017/s1462399410001390 20211047

[B23] Liu X. Yu X. Zack D. J. Zhu H. Qian J. (2008). TiGER: a Database for Tissue-specific Gene Expression and Regulation. BMC Bioinformatics 9, 271. 10.1186/1471-2105-9-271 18541026 PMC2438328

[B24] MacDonald J. R. Ziman R. Yuen R. K. C. Feuk L. Scherer S. W. (2014). The Database of Genomic Variants: a Curated Collection of Structural Variation in the Human Genome. Nucl. Acids Res. 42, D986–D992. 10.1093/nar/gkt958 24174537 PMC3965079

[B25] Makino T. McLysaght A. Kawata M. (2013). Genome-wide Deserts for Copy Number Variation in Vertebrates. Nat. Commun. 4, 2283. 10.1038/ncomms3283 23917329

[B26] Mao J. Wang H. Li H. Song X. Wang T. Xiang J. (2019). Genetic Analysis of Products of conception Using a HLPA/SNP-array Strategy. Mol. Cytogenet. 12, 40. 10.1186/s13039-019-0452-2 31687045 PMC6822274

[B27] Orellana C. Roselló M. Monfort S. Mayo S. Oltra S. Martínez F. (2015). Pure Duplication of 19p13.3 in Three Members of a Family with Intellectual Disability and Literature Review. Definition of a New Microduplication Syndrome. Am. J. Med. Genet. 167, 1614–1620. 10.1002/ajmg.a.37046 25858326

[B28] Palumbo P. Palumbo O. Leone M. P. Stallone R. Palladino T. Zelante L. (2016). Clinical and Molecular Characterization of a De Novo 19p13.3 Microdeletion. Mol. Cytogenet. 9, 40. 10.1186/s13039-016-0252-x 27239227 PMC4882821

[B29] Parchem J. G. Sparks T. N. Gosnell K. Norton M. E. (2018). Utility of Chromosomal Microarray in Anomalous Fetuses. Prenatal Diagn. 38, 140–147. 10.1002/pd.5202 PMC5828907 29297200

[B30] Popowski M. Lee B. K. Rhee C. Iyer V. R. Tucker H. O. (2017). Arid3a Regulates Mesoderm Differentiation in Mouse Embryonic Stem Cells. J. Stem Cel Ther Transpl. 1, 52–62. 10.29328/journal.jsctt.1001005 PMC6510499 31080945

[B31] Practice Committee of the American Society for Reproductive Medicine (2012). Evaluation and Treatment of Recurrent Pregnancy Loss: a Committee Opinion. Fertil. Steril 98, 1103–1111. 10.1016/j.fertnstert.2012.06.048 22835448

[B32] Qi H. Xuan Z.-L. Du Y. Cai L.-R. Zhang H. Wen X.-H. (2018). High Resolution Global Chromosomal Aberrations from Spontaneous Miscarriages Revealed by Low Coverage Whole Genome Sequencing. Eur. J. Obstet. Gynecol. Reprod. Biol. 224, 21–28. 10.1016/j.ejogrb.2018.03.008 29525519

[B33] Quintana-Murci L. Krausz C. McElreavey K. (2001). The Human Y Chromosome: Function, Evolution and Disease. Forensic Sci. Int. 118, 169–181. 10.1016/s0379-0738(01)00387-5 11311832

[B34] Rajcan-Separovic E. Diego-Alvarez D. Robinson W. P. Tyson C. Qiao Y. Harvard C. (2010). Identification of Copy Number Variants in Miscarriages from Couples with Idiopathic Recurrent Pregnancy Loss. Hum. Reprod. 25, 2913–2922. 10.1093/humrep/deq202 20847186

[B35] Reddy U. M. Page G. P. Saade G. R. Silver R. M. Thorsten V. R. Parker C. B. (2012). Karyotype versus Microarray Testing for Genetic Abnormalities after Stillbirth. N. Engl. J. Med. 367, 2185–2193. 10.1056/nejmoa1201569 23215556 PMC4295117

[B36] Redon R. Ishikawa S. Fitch K. R. Feuk L. Perry G. H. Andrews T. D. (2006). Global Variation in Copy Number in the Human Genome. Nature 444, 444–454. 10.1038/nature05329 17122850 PMC2669898

[B37] Rhee C. Lee B. K. Beck S. Anjum A. Cook K. R. Popowski M. (2015). Corrigendum: Arid3a Is Essential to Execution of the First Cell Fate Decision via Direct Embryonic and Extraembryonic Transcriptional Regulation. Genes Dev. 29, 1890. 10.1101/gad.247163.114 26341560 PMC4573860

[B38] Riggs E. R. Andersen E. F. Cherry A. M. Kantarci S. Kearney H. Patel A. (2020). Technical Standards for the Interpretation and Reporting of Constitutional Copy-Number Variants: a Joint Consensus Recommendation of the American College of Medical Genetics and Genomics (ACMG) and the Clinical Genome Resource (ClinGen). Genet. Med. 22, 245–257. 10.1038/s41436-019-0686-8 31690835 PMC7313390

[B39] Rosenfeld J. A. Tucker M. E. Escobar L. F. Neill N. J. Torchia B. S. McDaniel L. D. (2015). Diagnostic Utility of Microarray Testing in Pregnancy Loss. Ultrasound Obstet. Gynecol. 46, 478–486. 10.1002/uog.14866 25846569

[B40] Sahlin E. Gustavsson P. Liedén A. Papadogiannakis N. Bjäreborn L. Pettersson K. (2014). Molecular and Cytogenetic Analysis in Stillbirth: Results from 481 Consecutive Cases. Fetal Diagn. Ther. 36, 326–332. 10.1159/000361017 25059832

[B41] Sato T. Migita O. Hata H. Okamoto A. Hata K. (2019). Analysis of Chromosome Microstructures in Products of conception Associated with Recurrent Miscarriage. Reprod. BioMedicine Online 38, 787–795. 10.1016/j.rbmo.2018.12.010 30926177

[B42] Schlegel P. N. (2002). The Y Chromosome. Reprod. BioMedicine Online 5, 22–25. 10.1016/s1472-6483(10)61592-1 12470541

[B43] Shimokawa O. Harada N. Miyake N. Satoh K. Mizuguchi T. Niikawa N. (2006). Array Comparative Genomic Hybridization Analysis in First- Trimester Spontaneous Abortions with 'normal' Karyotypes. Am. J. Med. Genet. A. 140, 1931–1935. 10.1002/ajmg.a.31421 16906550

[B44] Suzuki N. Hirano K. Ogino H. Ochi H. (2019). Arid3a Regulates Nephric Tubule Regeneration via Evolutionarily Conserved Regeneration Signal-Response Enhancers. Elife 8, e43186. 10.7554/eLife.43186 30616715 PMC6324879

[B45] Uddin M. Tammimies K. Pellecchia G. Alipanahi B. Hu P. Wang Z. (2014). Brain-expressed Exons under Purifying Selection Are Enriched for De Novo Mutations in Autism Spectrum Disorder. Nat. Genet. 46, 742–747. 10.1038/ng.2980 24859339

[B46] Wang B. T. Chong T. P. Boyar F. Z. Kopita K. A. Ross L. P. El-Naggar M. M. (2014). Abnormalities in Spontaneous Abortions Detected by G-Banding and Chromosomal Microarray Analysis (CMA) at a National Reference Laboratory. Mol. Cytogenet. 7, 33. 10.1186/1755-8166-7-33 24914406 PMC4049495

[B47] Wang Y. Cheng Q. Meng L. Luo C. Hu H. Zhang J. (2017). Clinical Application of SNP Array Analysis in First-Trimester Pregnancy Loss: a Prospective Study. Clin. Genet. 91, 849–858. 10.1111/cge.12926 27883173

[B48] Wang Y. Li Y. Chen Y. Zhou R. Sang Z. Meng L. (2020). Systematic Analysis of Copy‐number Variations Associated with Early Pregnancy Loss. Ultrasound Obstet. Gynecol. 55, 96–104. 10.1002/uog.20412 31364215

[B49] Wapner R. J. Martin C. L. Levy B. Ballif B. C. Eng C. M. Zachary J. M. (2012). Chromosomal Microarray versus Karyotyping for Prenatal Diagnosis. N. Engl. J. Med. 367, 2175–2184. 10.1056/nejmoa1203382 23215555 PMC3549418

[B50] Wilcox A. J. Weinberg C. R. O'Connor J. F. Baird D. D. Schlatterer J. P. Canfield R. E. (1988). Incidence of Early Loss of Pregnancy. N. Engl. J. Med. 319, 189–194. 10.1056/nejm198807283190401 3393170

[B51] Witt M. (2019). Anatomy and Development of the Human Taste System. Handb Clin. Neurol. 164, 147–171. 10.1016/b978-0-444-63855-7.00010-1 31604544

[B52] Xie J. Xu Y. Wan L. Wang P. Wang M. Dong M. (2018). Involvement of Follistatin-like 3 in Preeclampsia. Biochem. Biophysical Res. Commun. 506, 692–697. 10.1016/j.bbrc.2018.10.139 30454705

[B53] Xu Y. Xie J. Wan L. Wang M. Xu Y. Wang H. (2020). Follistatin-like 3, an Activin A Binding Protein, Is Involved in Early Pregnancy Loss. Biomed. Pharmacother. 121, 109577. 10.1016/j.biopha.2019.109577 31810141

[B54] Yamada H. Sata F. Saijo Y. Kishi R. Minakami H. (2005). Genetic Factors in Fetal Growth Restriction and Miscarriage. Semin. Thromb. Hemost. 31, 334–345. 10.1055/s-2005-872441 16052406

[B55] Yang Y. Qu S. Wang L. Guo Y. Xue S. Cai A. (2019). Genetic Testing of Chorionic Villi from Abortuses during Early Pregnancy. Zhonghua Yi Xue Yi Chuan Xue Za Zhi 36, 547–551. 10.3760/cma.j.issn.1003-9406.2019.06.004 31055802

[B56] Zarrei M. MacDonald J. R. Merico D. Scherer S. W. (2015). A Copy Number Variation Map of the Human Genome. Nat. Rev. Genet. 16, 172–183. 10.1038/nrg3871 25645873

[B57] Zhang W. Lei T. Fu F. Deng Q. Li R. Wang D. (2021). Microarray Analysis in Fetuses with Duodenal Obstruction: It Is Not Just Trisomy 21. Prenatal Diagn. 41, 316–322. 10.1002/pd.5834 33000500

[B58] Zhu X. Li J. Zhu Y. Wang W. Wu X. Yang Y. (2018). Application of Chromosomal Microarray Analysis in Products of Miscarriage. Mol. Cytogenet. 11, 44. 10.1186/s13039-018-0396-y 30140311 PMC6098645

[B59] Zinaman M. J. Clegg E. D. Brown C. C. O’Connor J. Selevan S. G. (1996). Estimates of Human Fertility and Pregnancy loss*†*Supported by grant CR-820787 from the United States Environmental Protection Agency, Washington, D.C.†The Views Expressed in This Paper Are Those of the Authors and Do Not Necessarily Reflect the Views or Policies of the U.S. Environmental Protection Agency. The U.S. Government Has the Right to Retain a Nonexclusive Royalty-free License in and to Any Copyright Covering This Paper. Fertil. Sterility 65, 503–509. 10.1016/s0015-0282(16)58144-8

