# Supplementary material for: Copy Number Variation Analysis of Euploid Pregnancy Loss
Source: Front Genet. 2022 Mar 23;13:766492. doi: 10.3389/fgene.2022.766492 (PMC8984164; doi:10.3389/fgene.2022.766492)
Supplement: Supplementary file 3 [file Table1.DOCX]

**Table S1** Complete search used in the systematic literature search

The following terms were used: fetal OR fetus OR product of conception, AND miscarriage OR fetal loss OR fetal demise OR fetal death OR pregnancy loss OR spontaneous abortion, AND, microarray OR array OR array-cgh OR cma OR chromosomal microarray analysis OR next-generation sequencing OR copy number variants, without any limitation applied. The studies included in the meta-analyses were also manually searched for further relevant studies.
