# Supplementary figures and images for: Copy Number Variation Analysis of Euploid Pregnancy Loss
Source: Front Genet. 2022 Mar 23;13:766492. doi: 10.3389/fgene.2022.766492 (PMC8984164; doi:10.3389/fgene.2022.766492)

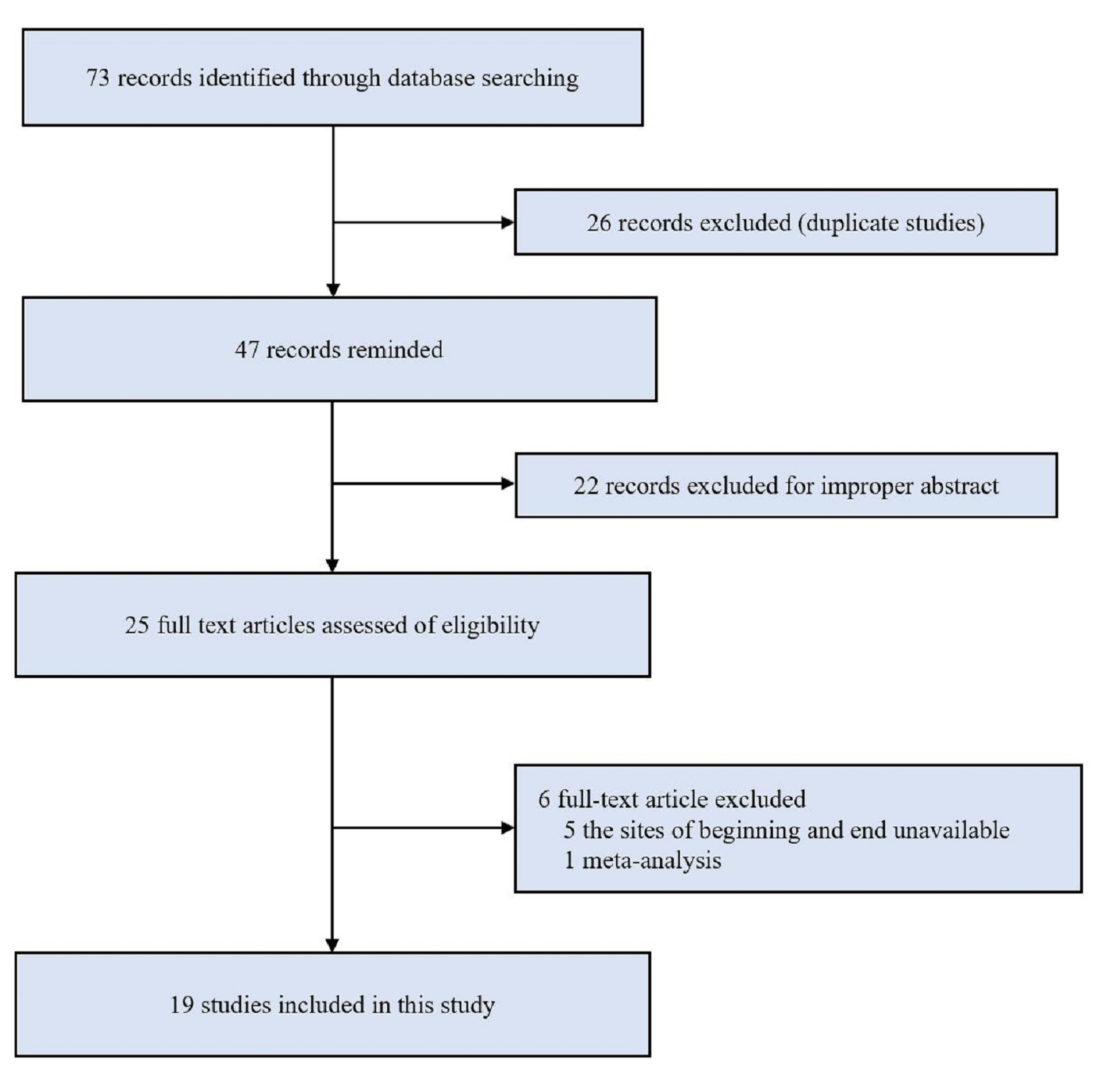

Supplement: Supplementary file 4 [file Image1.TIF]

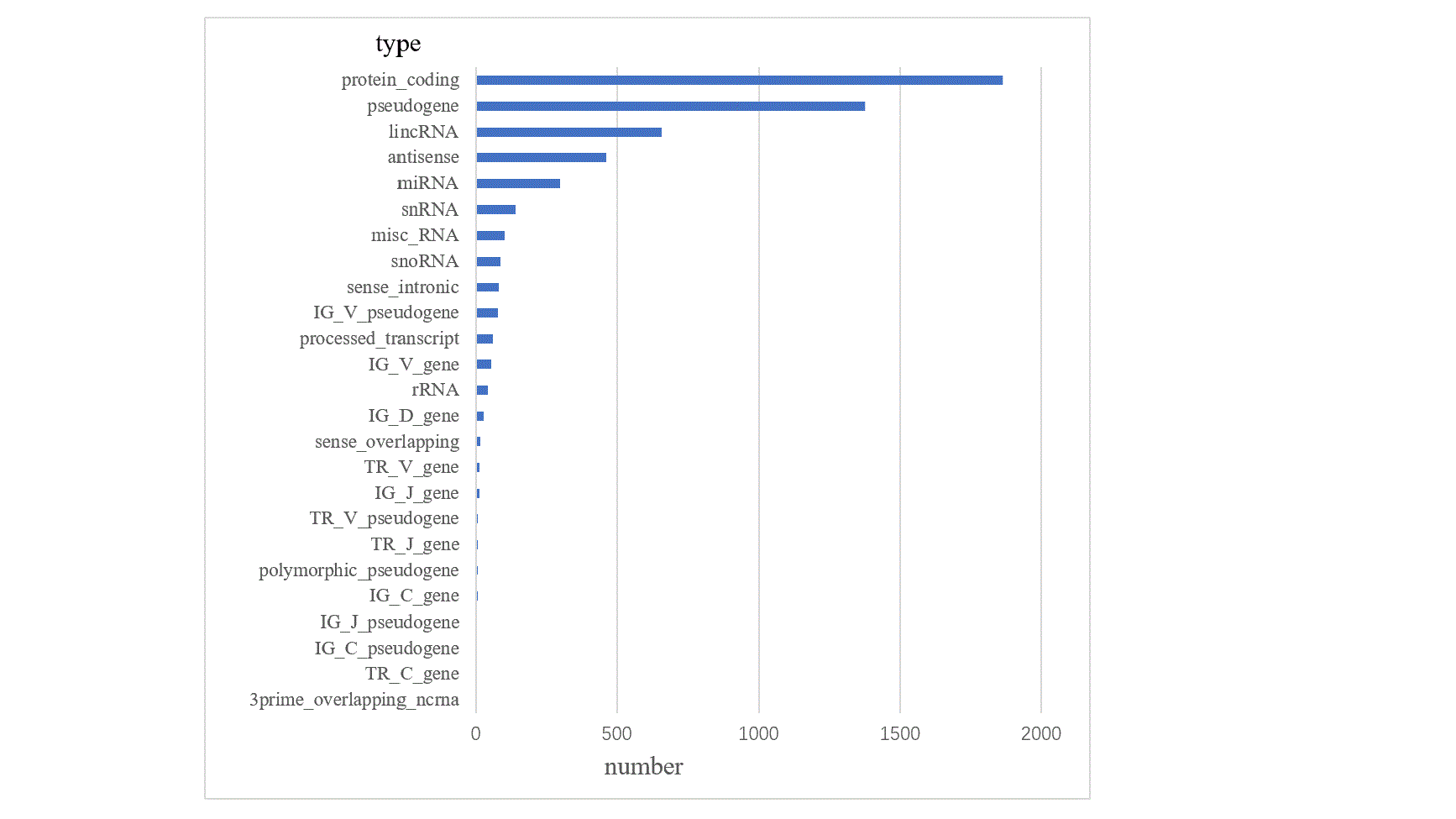

Supplement: Supplementary file 5 [file Image2.TIFF]
